# Local Government Competition, Environmental Regulation Intensity and Regional Innovation Performance: An Empirical Investigation of Chinese Provinces

**DOI:** 10.3390/ijerph16122130

**Published:** 2019-06-16

**Authors:** Jinqian Deng, Na Zhang, Fayyaz Ahmad, Muhammad Umar Draz

**Affiliations:** 1 School of Economics, Lanzhou University, Lanzhou 730000, China; dengjq@lzu.edu.cn (J.D.); fayyaz@lzu.edu.cn (F.A.); 2 Department of Management and Humanities, Universiti Teknologi PETRONAS, 32610 Seri Iskandar, Malaysia; umar.draz@utp.edu.my

**Keywords:** local government competition, environmental regulation intensity, regional innovation performance

## Abstract

The aim of this paper is to examine the impact of local government competition and environmental regulation intensity on regional innovation performance and its regional heterogeneity. Based on the theoretical mechanism of the aforementioned variables, this study uses the Chinese provincial panel data from 2001 to 2016. We use the super-efficiency data envelopment analysis (SE-DEA) to evaluate regional innovation performance. To systematically examine the impact of local government competition and environmental regulation intensity on regional innovation performance, we build a panel date model using the feasible generalized least squares (FGLS) method. The results indicate that: the regional innovation performance can be significantly improved through technological spillover; local governments compete for foreign direct investment (FDI) to participate in regional innovative production. Moreover, improvements in environmental regulation intensity enhance regional innovation performance through the innovation compensation effect. Our results show that the local governments tend to choose lower environmental regulation intensity to compete for more FDI, which has an inhibitory effect on regional innovation performance. Furthermore, due to regional differences in factor endowments, economic reforms and economic development levels in Chinese provinces, there exists a significant regional consistency in the impact of local government competition and environmental regulation intensity on regional innovation performance. Therefore, institutional arrangements and incentive constraints must be adopted to enhance regional innovation performance as well as to guide and foster the mechanism of green innovation competition among local governments. At the same time, considering the regional heterogeneity of local government competition and environmental regulation intensity affecting regional innovation performance, policy makers should avoid the “one-size-fits-all” strategy of institutional arrangements.

## 1. Introduction

National innovation system is an innovation network which is organically combined with the regional innovation system within a country, and research on innovation performance remains a fundamental area of economic analysis [1]. Within the overall framework of the national innovation system, the regional innovation system must consider the intensity of regional environmental regulation when organizing and competing innovation factors for innovative production. Therefore, how to improve the performance of regional innovation by organizing innovation factors under the condition of environmental constraints is a topic worthy of in-depth study. In the case of the Chinese economy, since 1978 it has created a “China miracle” in the history of world economic growth driven by “factor input”. Due to environmental and resource constraints coupled with the decline of demographic dividend, the drawbacks and unsustainability of the factor input-driven growth model are increasingly evident. Therefore, the steady-state transformation of China’s economic structure needs to find new power sources. In the process of regional innovation in China, the role of local governments has become the most important factor in the regional innovation system. Local governments influence regional innovation performance by competing for external innovation factors and choosing environmental regulation intensity. Chinese economic structural transition and steady transition of growth motivation in the new era is based on the principle of innovation-driven green development. The local governments should not only organize innovative elements within the region, but also actively compete for all possible innovative elements outside the region for innovative production and insist on green development. The essence of environmental regulation is to maintain the non-decreasing level of ecological capital stock [2]. Therefore, it is of great significance to study the impact of local government competition and environmental regulation intensity on regional innovation performance.

Compared to the existing research, the contribution of this paper is mainly reflected in the following two points: firstly, constructing a stepwise regression model to examine the impact of local government competition on regional innovation performance and environmental regulation intensity, and further analyzing the intermediary effect of local government competition on regional innovation performance through environmental regulation intensity; secondly, in the framework of empirical analysis, the interaction item of local government competition and environmental regulation intensity is included to test the impact of the synergy of both variables on regional innovation performance. The structure of this paper is as follows: after the introduction, the second part consists of literature reviews aiming to clarify the research context in this field; the third part presents the theoretical analysis framework aiming to analyze the mechanism of local government competition and environmental regulation intensity on regional innovation performance; the fourth part shows details of variables, data and empirical strategies; the fifth part comprises of empirical analyses and discussion of results; the sixth part presents the conclusion and policy recommendations of this paper.

## 2. Literature Review

In the regional innovation system, local governments are important actors. They not only organize innovative factors in the region to carry out innovative production, but also compete actively for all possible innovative factors outside the region to participate in innovative production in the jurisdiction. To achieve environmental and economic sustainability, environmental regulation is the government’s direct or indirect intervention in regional environmental pollution by means of economic or social regulation. Therefore, the impact of local government competition and environmental regulation intensity on regional innovation performance is complex. To address this intricate subject, the review of existing literature has been classified into different sections.

### 2.1. Local Government Competition and Regional Innovation Performance

The institutional arrangement of Chinese-style decentralization fosters yardstick competition among local governments through political and economic incentives [3]. Local government focuses on attracting liquidity factors in a fierce competition to attract capital, labor and other factors to serve regional development as much as possible [4]. Local governments launch yardstick competition focusing on “attract bid to invite investments”, especially competing to attract foreign direct investment [5]. Foreign Direct Investment (FDI) has a positive impact on the innovation efficiency of the host country because of its technology spillover [6,7]. Girma and Wakelin’s investigation [8] based on the UK electronics industry proves that FDI inflows can significantly enhance the innovation capability of host countries. Hu and Jefferson [9] also verify that FDI has a positive effect on the number of regional patent applications. Based on China’s provincial panel data, Sang and Yue [10] found that FDI technology spillover has a significant, positive effect on China’s independent innovation capability, however, there is significant regional heterogeneity in the eastern, central and western regions. Wang and Zhang [11] examined the heterogeneity of the impact of FDI and official cooperation of industries on regional innovation output; the authors found that FDI has a significant effect on the output of new products, but not on the output of patents. In the background of innovation-driven development strategic, fiscal decentralization reform encourages local governments to increase their support for technological innovation, as competition for innovation can significantly promote the efficiency of technological innovation [12].

### 2.2. Local Government Competition and Environmental Regulation Intensity

In the context of local government competition, there are also competing attributes of environmental regulation in different regions because of cross-regional capital competition and cross-border pollution. There is a “pollution heavens” hypothesis which suggests that high-income countries have higher environmental regulation intensity; enterprises’ environmental regulation costs are relatively high. The low-income countries tend to have lower environmental regulation intensity in order to attract foreign investment [13,14]. In view of the impact of local government competition on the environmental regulation intensity under Chinese decentralization, He et al. [15] confirmed that local government competition has negative environmental effects. Zhang [16] believes that under the influence of local government competition, environmental regulation has significantly promoted carbon emissions, triggering the phenomenon of “bottom-up effect” and “green paradox” in environmental regulation. Wang et al. [17] combined with the “pollution shelter” hypothesis, believed that local governments will reduce the level of environmental regulation in order to compete for more FDI. The game of environmental policy among local governments has the characteristics of the long-term competition. Through empirical research based on data from prefecture-level cities, Zhu et al. [18] found that local government competition can increase environmental pollution in local and surrounding areas and the high intensity of local environmental regulation will lead to the aggravation of pollution in the surrounding areas.

### 2.3. Environmental Regulation Intensity and Regional Innovation Performance

Government environmental regulation aims at maintaining non-reduction of environmental policy. Porter [19] put forward the "Porter hypothesis" after a dynamic investigation of environmental regulation policy, believing that environmental regulation policy will produce innovation compensation effect and achieve a “win-win” state of environment and innovation. After that, many scholars have carried out a lot of empirical studies on the relationship between environmental regulation and technological innovation, For instance, Hamamoto’s study [20] on Japan proved that with the increase of investment expenditure on environmental pollution control, the corresponding R&D expenditure will also increase, which has a significant impact on improving technological innovation capability. Liu and Yan [21] found that environmental regulation has a significant positive impact on invention patents and utility model patents, and there is a lag in this impact, but the impact on design patents is not significant. Furthermore, there are significant regional differences in the impact of environmental regulation on regional innovation. Shen [22] found that the impact of environmental regulation intensity on technological innovation is non-linear and the relationship between them is in line with the “U” type. This indicates that the impact of the enhancement of environmental regulation intensity on technological innovation first decreases and then increases, and it can be explained from the perspective of path dependence of technological progress [23], and it’s closely related to regional or corporate endowments and geographical location [24]. Qi and Tao [25] used the GWR model to explore the impact of factor agglomeration on regional innovation capability under environmental constraints. They found that environmental regulation has a dual impact on regional innovation capability.

To summarize, many scholars have explored the relationship between local government competition, environmental regulation intensity and regional innovation performance from multiple perspectives, which provides theoretical reference and logical starting point for this study. However, through reviewing the literature, it is clear that there is nothing that incorporates local government competition, environmental regulation intensity and regional innovation performance into a systematic analysis framework, and this association and its impact needs further investigation. This paper makes an empirical study with comprehensive FGLS based on the panel data of Chinese provinces from 2001 to 2016, in order to make the conclusion more authentic and reliable.

## 3. Theoretical Analysis Framework

According to the theory of national innovation system, this system is an organic combination of a regional innovation system. It includes not only the innovation production activities of the main bodies in a regional innovation system, but also the competition relationship among regional innovation systems [26]. Therefore, within the overall framework of the national innovation system, only by considering the competition relationship between innovators and the environmental regulation policy in the regional innovation system, can we reveal the organizational mode of the elements of the regional innovation system in a more comprehensive way, then provide useful reference for improving regional innovation performance and national innovation performance level [27]. This section will systematically elaborate the mechanism of the impact of local government competition and environmental regulation intensity on regional innovation performance.

### 3.1. Analysis on the Mechanism of the Impact of Local Government Competition on Regional Innovation Performance

Based on the analysis of the existing literature and the actual situation, local government competition for FDI has a dual effect on regional innovation performance. Firstly, we realize the promotion effect. In the regional innovation system, local governments compete for FDI to participate in innovative production, which can not only make up for the shortage of funds in the regional innovation system, increase the input of elements in the regional innovation system, but also activate the existing resources and stock, have a positive effect on the regional innovation performance. Additionally, the competitive FDI of local governments often has the advantages of technology and management, which is an important carrier of technology spillover. Through demonstration-imitation effect, competition effect, linkage effect and personnel training and mobility effect of transnational corporations, regional technological level and organizational efficiency are constantly improved [28]. Secondly, we notice the restraint effect. Local government competition for FDI will aggravate the competition intensity of regional market, lead to the reduction of profits of original enterprises, even crowding out the original enterprises, produce “crowding-out effect” on innovation activities in the region, and cause the shrinkage of innovation market and the decline of innovation output. Additionally, in order to maintain its competitive advantage, FDI often suppresses local competitors and blockades technology spillover by setting barriers, which has a restraining effect on the improvement of regional innovation performance.

### 3.2. Analysis on the Mechanism of the Impact of Local Government Competition on Environmental Regulation

Porter’s “pollution shelter” hypothesis holds that local government’s environmental regulation policy will lead to an increase in the “compliance cost” of enterprise production, and enterprises will choose lower standards of environmental regulation areas to invest in order to gain competitive advantage, then forming a “pollution shelter”. In order to gain competitive advantage in the process of competition for foreign direct investment, local governments often adopt fiscal compensation as tax incentives, even regard environmental policy as an important means of competition. When the surrounding areas reduce environmental standards and regulatory levels in order to attract FDI, the region will adopt similar competitive strategies to attract more investment from polluting enterprises by reducing the level of environmental regulation. Therefore, local government competition has a restraining effect on the intensity of environmental regulation. However, in the long run, “pollution shelter” has become a disadvantage of local governments, which is not conducive to attracting FDI. With the practice of decentralized governance and ecological civilization construction in China, local governments will effectively implement the central government’s environmental policies and increase investment in environmental governance, enhance the intensity of environmental regulation, and improve the regional ecological environment to maintain competitive advantage.

### 3.3. Analysis on the Mechanism of the Impact of Environmental Regulation Intensity on Regional Innovation Performance

Neoclassical economic theory holds that environmental protection will enhance the overall welfare of society, but the government’s environmental regulation policy will increase the “compliance cost” of enterprises, which seriously hinders the improvement of productivity and innovation ability of enterprises. But the “Porter Hypothesis” systematically expounds the possibility of “win-win” between environmental protection and enterprise innovation. Facing the strict environmental regulation intensity of the government, enterprises will control pollutant emissions in the following two ways: Firstly, the “effect of technological progress in pollution control” of enterprises means that enterprises reduce pollution levels through expenditure on pollution control; Secondly, the “innovation compensation effect” of enterprises, which means that enterprises ultimately reduce or offset the increased “compliance cost” of government environmental regulation through technological improvement. Therefore, the improvement of production technology and innovation compensation can pay for the cost of pollution treatment, and then bring about environmental regulation and enterprise innovation to achieve a “win-win”. In addition, environmental regulation will also have a negative impact on innovation through the “crowding-out effect of innovation funds” and the “crowding-out effect of investment”. On the one hand, higher intensity of environmental regulation will increase the cost of pollution control of enterprises, but under the condition of fixed total resources, increasing investment in environmental governance will inevitably lead to the reduction of innovation investment, and the enhancement of intensity of environmental regulation will inhibit the improvement of regional innovation performance; On the other hand, the intensity of strict environmental regulation will lead to the withdrawal of enterprises, the re-allocation of production and investment in areas with low regulation level, and the reduction of local investment and share of innovation investment, which will affect regional innovation performance.

**Figure 1:**
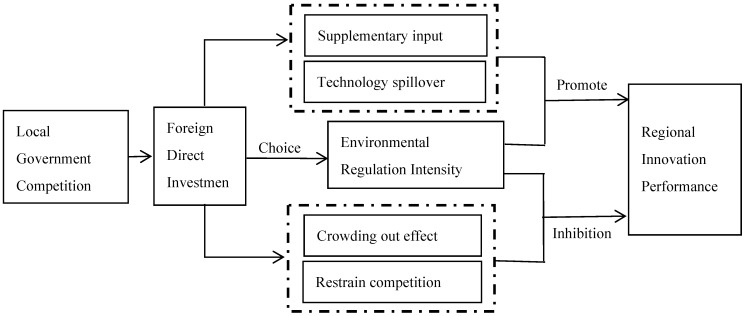
The influence mechanism of local government competition and environmental regulation intensity on regional Innovation performance.

To sum up, the impact of local government competition and environmental regulation intensity on regional innovation performance is twofold. In order to better identify the mechanism of impact, we draw the theoretical framework in Figure 1. The specific impact effects shown in this framework will be empirically examined through empirical models.

## 4. Variables, Data and Empirical Strategies

### 4.1. Variable Definition and Explanation

#### 4.1.1. Dependent Variable: Regional Innovation Performance

The number of patent authorizations is usually selected to evaluate regional innovation performance in existing literature. The patent data is easy to obtain but it is controversial because it cannot measure the quality of innovation output and the level of market application. Therefore, some scholars began to use sales revenue of new products as the proxy variable of innovation performance [29]. Based on Wang and Huang [30] approach, this paper chooses the SE-DEA model to evaluate regional innovation performance.

Suppose there are k decision-making units, i.e. provincial units in this paper, (k = 1, 2, 3……K) and each decision-making unit uses n (n = 1, 2, 3……N) innovative factors to put into production. To get m kinds of innovation output (m = 1, 2, 3……M), the following SE-DEA model is constructed shown in Equation (1):

*Minimize*θ
*subjectto*$\sum_{\begin{array}{l} j=1\\ j\neq 0 \end{array}}^{k}x_{ij}\lambda_{j}\leq x_{i0},i=1,2,\cdots n$
  (1) $$\sum_{\begin{array}{l} j=1\\ j\neq 0 \end{array}}^{k}y_{rj}\lambda_{j}\geq y_{r0},r=1,2,\cdots m\lambda_{i}\geq 0,j\neq 0$$

Among them, θ is the innovation efficiency of each decision-making unit, $x_{io}$ and $y_{ro}$ are the input of innovation elements and output indicators of innovation products in decision-making units, $x_{ij}$ and $y_{rj}$ represent input column vectors of innovation elements and output column vectors of innovation products in decision making units, and $\lambda_{j}$ denotes combination coefficients.

**Figure 2:**
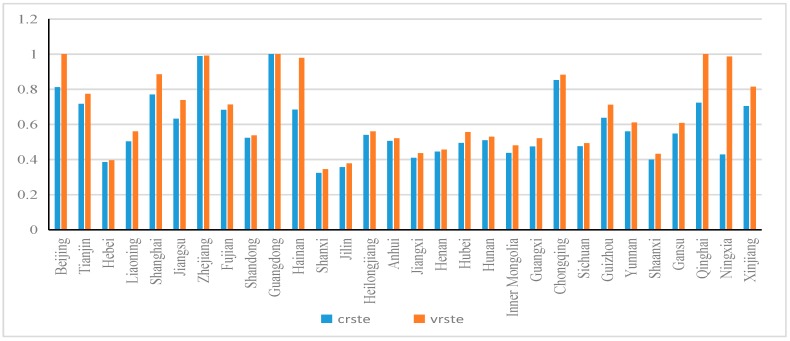
Average Value of Innovation Performance of Provincial Units during the Investigation Period.

It can be observed that the basics of regional innovation performance evaluation are to determine the input of regional innovation factors and the output of innovative products. R&D plays a key role in the input of innovation factors, and its effect in improving the success rate of innovation has been recognized by the academic circles [31]. According to the research in this paper, the input of innovation factors is divided into two indicators: R&D personnel and R&D capital. The R&D personnel input index adopts the full-time equivalent of R&D personnel in each region during the sample period. The index of R&D capital input is represented by R&D capital stock according to Aldieri et al. [32]. The output of innovative products is estimated by two indicators: the number of patents authorized by each region and the turnover of new technology market in the sample period. Among them, the number of patents authorized represents the effective output of regional innovative products. Whereas, the technology market turnover represents the transformation and marketization of innovative products. All the data are taken from the China Statistical Yearbook and the China Science and Technology Statistical Yearbook from 2001–2016. This study uses the data of 30 provinces and the Tibet is excluded due to data unavailability. Figure 2 reports the average value of regional innovation performance in the period of investigation; in which crste is the average value without considering the measurement of returns to scale, and vrste is the average value with regard to the measurement of return on scale. In our empirical analyses, the vrste index is used.

#### 4.1.2. Core Explanatory Variables

The local governments compete as much as possible for innovation factors outside the region to participate in innovation activities in the regional innovation system. The dominant strategy of attracting FDI has become the concentrated reflection of local government competition. Therefore, this paper uses the logarithmic value of regional per capita actual utilization of FDI to calculate local government competition; the greater the actual amount of FDI per capita in a region, the stronger the competitiveness of local governments in the region. The data is taken from China Statistical Yearbook.

The second main variable is the environmental regulation intensity (er). There are many methods to calculate the intensity of environmental regulation: firstly, evaluating the policy quantity of environmental regulation. Secondly, assessment based on contaminated income finally, the ratio of expenditure on pollution treatment and control to production cost or GDP is used to evaluate the proxy. In this paper, the evaluation of environmental regulation intensity focuses on investment in environmental pollution control, selecting the ratio of total investment in regional environmental pollution control to GDP to measure regional environmental regulation intensity. The data is collected from China Environmental Statistics Yearbook.

#### 4.1.3. Control Variables

In order to accurately reveal the impact of local government competition and environmental regulation intensity on regional innovation performance and obtain more robust estimation results, other relevant factors affecting regional innovation performance must be controlled as far as possible. Referring to the existing research literature, this paper chooses government intervention, non-agricultural industry development, urbanization rate, financial development, human capital stock level and the degree of opening as control variables. The specific definitions are shown in Table 1. The data are collected from China Statistical Yearbook, China Population Employment Statistics Yearbook and the statistical yearbooks of all provinces (autonomous regions and municipalities directly under the Central Government) from 2002 to 2017.

### 4.2. Method

Considering the problem of heteroscedasticity and correlation in ordinary least squares (OLS), in this paper, the feasible generalized least squares method (FGLS) is used. It is based on the effective transformation of generalized least squares (GLS) [33].

Firstly, the formula of OLS is transformed as shown in Equation (2):

(2) ϕY=ϕXβ+ϕV

where $\phi V\underline{\underline{\Delta }}\nu$ satisfies the requirement of OLS method for error terms.

Secondly, the OLS estimation of (2) is carried out, and the estimated value is given in Equation (3):

(3) $$\hat{\beta }={\left(X^{T}\phi^{T}\phi X\right)}^{-1}X^{T}\phi^{T}\phi Y$$

Therefore, $\hat{\beta }-\beta ={\left(X^{T}\phi^{T}\phi X\right)}^{-1}X^{T}\phi^{T}\phi V={\left(X^{T}\phi^{T}\phi X\right)}^{-1}X^{T}\phi^{T}\nu$.

If $E(X^{T}\nu )=EX^{T}E\nu =X^{T}E\nu$ holds, the estimated value $\hat{\beta }$ obeys normal distribution with expectation and variance, it is describe in Equation (4) and Equation (5) below:

(4) $$E\hat{\beta }=\beta$$

(5) $$Var\hat{\beta }={\hat{\sigma }}_{\nu }^{2}{\left(X^{T}\phi^{T}\phi X\right)}^{-1}$$

In the above equation:

(6) $$\begin{array}{cc} {\hat{\sigma }}_{\nu }^{2}=S^{2} & =\frac{1}{\left(n-2\right)}{\left(\phi Y-\phi X\hat{\beta }\right)}^{T}(\phi Y-\phi X\hat{\beta })\\ & =\frac{1}{\left(n-2\right)}[Y^{T}\phi^{T}\phi Y+Y^{T}\phi^{T}\phi X{\left(X^{T}\phi^{T}\phi X\right)}^{-1}X^{T}\phi^{T}\phi Y] \end{array}$$

In FGLS estimation, the transformation matrix ϕ is unknown. It must be estimated first to get $\hat{\phi }$, and then to get β in Equation (2). Generally, the estimated value can reach the expected accuracy in repeated iterations.

### 4.3. Econometric Model

Based on the above analysis, this paper attempts to make an empirical study on the relationship between local government competition and environmental regulation intensity affecting regional innovation performance. In order to test the mediating effect of environmental regulation intensity on regional innovation performance, a stepwise regression model was designed based on existing research [34]. At first, in order to test the impact of local government competition on regional innovation performance without considering the intensity of environmental regulation and other factors affecting regional innovation performance, this paper sets the following model shown in Equation (7):

(7) $$\text{Model 1:}\;\theta_{it}=c_{0}+c_{1}fdi_{it}+\mu_{i}+\gamma_{t}+\vartheta_{it}$$

Among them, i represents provincial units, t representation time unit, θ represents regional innovation performance,fdi represents local government competition, $\mu_{i}$ represents individual fixation effect, $\gamma_{t}$ represents time-fixed effect, ϑ is a residual term. It is assumed that white noise sequence is obeyed; $c_{0}$ and $c_{1}$ is the parameter to be estimated.

Secondly, in order to examine the indirect effect of environmental regulation intensity on regional innovation performance in local government competition, it is divided into two steps to construct the following two models, formulas are given in Equation (8) and (9):

(8) $$\text{Model 2:}\;er_{it}=\beta_{0}+\beta_{1}fdi_{it}+\mu_{i}+\gamma_{t}+\varepsilon_{it}$$

(9) $$\text{Model 3:}\;\theta_{it}=\eta_{0}+\eta_{1}fdi_{it}+\eta_{2}er_{it}+\eta_{3}fdi_{it}\times er+\mu_{i}+\gamma_{t}+\omega_{it}$$

Among them, Model 2 takes the intensity of environmental regulation as the explanatory variable, local government competition as explanatory variable, to test the impact of local government competition on the intensity of environmental regulation. Model 3 takes regional innovation performance as the explanatory variable, local government competition, environmental regulation intensity and their interaction terms are the core explanatory variables to test the direct and indirect effects of local government competition and environmental regulation intensity on regional innovation performance.

Finally, in order to accurately test the impact of local government competition and environmental regulation intensity on regional innovation performance, other factors affecting regional innovation performance must be controlled as far as possible. Referring to the existing research and control variable design, model 4 is constructed:

(10) $$\text{Model 4:}\;\theta_{it}=\phi_{0}+\phi_{1}fdi_{it}+\phi_{2}er_{it}+\phi_{3}fdi_{it}\times er+\sum_{j=1}\lambda_{j}X_{ijt}+\mu_{i}+\gamma_{t}+\omega_{it}$$

Here, $X_{ijt}$ is a set of control variables, the specific settings are shown in Table 1.

## 5. Empirical Results and Discussion

### 5.1. Benchmark Estimation Results and Discussion

According to the description of the empirical strategies mentioned above, the FGLS is selected to estimate model 1 to 4. The estimated results are shown in Table 2.

The results of model 1 show that the coefficient of influence of local government competition on regional innovation performance is 0.052 and it has statistical significance at the level of 1%. This indicates that local government competition can significantly improve regional innovation performance. On the one hand, local governments compete for FDI to participate in regional innovation production, which makes up for the shortage of funds for the operation of the regional innovation system, stimulates innovation activities of various innovators, and improves the performance of regional innovation. On the other hand, local governments compete for FDI which produces technology spillovers in investment areas because of its comparative advantages in technology and management. Similarly, local innovators improve regional innovation performance through learning.

The result of model 2 illustrates that the coefficient of influence of local government competition on the intensity of environmental regulation is −0.145 and statistically significant indicating that local government competition will significantly reduce the intensity of environmental regulation. The possible reason is that local governments compete for growth under the Chinese decentralization system; they not only organize economic factors within their jurisdiction to pursue economic growth, but also actively compete for all possible economic factors to serve the economic growth of their jurisdiction. Therefore, local governments tend to choose lower environmental regulation intensity in order to attract more FDI.

In order to test whether local government competition can influence regional innovation performance through the intensity of environmental regulation, model 3 is estimated. The results show that the competition of local governments and the intensity of environmental regulation have positive effects on improving regional innovation performance. The environmental regulation will produce innovation compensation effect and innovation investment will also increase with the enhancement of environmental regulation intensity. The estimated coefficient of the interactive term between local government competition and environmental regulation intensity is significantly negative, which indicates that local government competition has a negative impact on regional innovation performance through the choice of environmental regulation intensity. This conclusion needs to be further validated to consider the influential variables.

On the basis of model 3, the model 4 incorporates six control variables: government intervention (gov), non-agricultural industry development (fn), urbanization rate (urb), financial development (fis), human capital stock (hum) and openness to the outside world (ope). The results display that the coefficients of local government competition, environmental regulation intensity and their interaction are identical with model 3 and statistically significant. This confirms that the local government competition and environmental regulation intensity can significantly improve regional innovation performance, and local government competition will inhibit regional innovation performance by choosing the intensity of environmental regulation. In terms of control variables, the estimation coefficient of government intervention on regional innovation performance is significantly positive, that is, the government organizes regional innovation factors to carry out innovative production and improve regional innovation performance. The effect of non-agricultural industry development on regional innovation performance is significantly negative, indicating that non-agricultural industry development has a significant inhibition effect on the improvement of regional innovation performance. However, the promotion of regional innovation performance is not to choose to inhibit non-agricultural industries, but to promote the transformation of innovation achievements through the development of non-agricultural industries, and to enhance the level of commercialization of innovation achievements. The impact coefficient of urbanization on regional innovation performance is positive, but it has no statistical significance. The impact of financial development on regional innovation performance is significantly positive, showing that the regional financial development can significantly improve regional innovation performance. The impact of human capital stock level on regional innovation performance is significantly negative, indicating that the stock of human capital does not improve the performance of regional innovation. The possible reason is that human capital allocation is distorted, and human resource dividend cannot be released. Likewise, opening to the outside world has a significantly positive effect on regional innovation performance through technology spillovers.

### 5.2. Robustness Test Results and Discussion

The results of benchmark estimation may be unstable because of the selection bias of interpreted and interpreted variables. Thus, in order to test the robustness of model settings and benchmark estimates, the four models are estimated by replacing the interpreted variables and the core explanatory variables. Table 3 reports the estimated results of replacing the interpreted variables and interpreted variables select the logarithmic value of new technology market turnover. This index reflects the marketization level of innovation achievements and it can represent regional innovation performance. Table 4 reports the estimated results of the replacement of core explanatory variables indicating that the proportion of the actual utilization of FDI in provinces to the actual utilization of FDI in the whole country is used as the agent variable of local government competition. The higher the ratio represents the stronger the competitiveness of local governments.

As can be perceived from Table 3 and Table 4, after transforming the interpreted variables and the core explanatory variables, the estimated coefficient symbols of model 1 to 4 are identical with the baseline regression, but the level of significance is different. The results show that the benchmark estimation results are robust to some extent.

### 5.3. Regional Heterogeneity Analysis of Sub-sample

Considering the regional differences of factor endowment, economic reform and economic development level in Chinese provinces and regions, this study further tests the regional heterogeneity of the impact of local government competition and environmental regulation intensity on regional innovation performance. Referring to the classification method of the mainstream literature, this paper divides the whole sample into three sub-samples: the eastern part, the central part and the western part. The eastern region includes 11 provincial administrative regions, including Beijing, Tianjin, Hebei, Liaoning, Shanghai, Jiangsu, Zhejiang, Fujian, Shandong, Guangdong and Hainan. The central region includes eight provincial administrative regions, including Shanxi, Jilin, Heilongjiang, Anhui, Jiangxi, Henan, Hubei and Hunan. The western region includes 11 provincial administrative regions, including Sichuan, Chongqing, Guizhou, Yunnan, Shaanxi, Gansu, Qinghai, Ningxia, Xinjiang, Guangxi and Inner Mongolia. Tibet is not included in the western region analyses because of the lack of data. Table 5 reports the estimated results of sub-regional sample models 3 and 4.

From the estimated results of the eastern samples, local governments’ competition for FDI can significantly improve regional innovation performance, and FDI has a significant technology spillover effect. The improvement of environmental regulation intensity also has a significant positive impact on regional innovation performance, while the interaction between local government competition and environmental regulation has a significant, negative impact on regional innovation performance. It shows that local government competition has a restraining effect on regional innovation performance through the choice of environmental regulation intensity, which is completely consistent with the estimated results of the national sample. From the estimated results of the central samples, local governments’ competition for FDI has a negative impact on regional innovation performance, which may be due to regional factor endowment differences. Local governments in central China compete for FDI, which has a crowding-out effect on regional innovation capability, without effective innovation compensation. Which makes it difficult for FDI technology to spillover, and the regional innovation performance cannot be effectively improved. The intensity of environmental regulation also inhibits the improvement of regional innovation performance in the central region; the possible reason is that local governments will choose a lower intensity of environmental regulation for innovation and economic growth. The interaction between local government competition and environmental regulation intensity has a significant, positive impact on regional innovation performance, indicating that local government competition will improve the regional innovation performance of the central region by choosing the intensity of environmental regulation. From the estimated results of the western samples, the estimated coefficients of local government competition are significantly different between model 3 and 4 showing that the impact of local government competition on regional innovation performance in the western region is inconsistent. Additionally, the intensity of environmental regulation can significantly improve the regional innovation performance of the western region. However, the interaction between local government competition and environmental regulation intensity has a significant, negative impact on regional innovation performance, which is consistent with the estimates of Eastern China. To summarize, the impact of local government competition and environmental regulation intensity on regional innovation performance has significant regional heterogeneity characteristics.

## 6. Conclusions and Policy Implications

The institutional arrangement of Chinese decentralization leads to local governments competing for growth; it also reduces the intensity of environmental regulation in order to gain the advantage of competitive FDI. In the process of promoting innovation-driven strategy and building an innovative China, local governments with competitive attributes are an important factor in the regional innovation system. Based on the theoretical mechanism of the impact of local government competition and environmental regulation intensity on regional innovation performance, this paper used the provincial panel data of China from 2001 to 2016. The super-efficiency SE-DEA model is employed to evaluate regional innovation performance. Furthermore, the FGLS method was used to examine the impact of local government competition and environmental regulation intensity on regional innovation performance and its regional heterogeneity. Our results showed that local governments compete for FDI to participate in regional innovative production, and regional innovation performance can be significantly improved through technology spillovers. Similarly, improvement of environmental regulation intensity can enhance regional innovation performance through innovation compensation effect. Moreover, in order to compete for more FDI, local governments tend to choose lower environmental regulation intensity, which has a restraining effect on regional innovation performance. Finally, the impact of local government competition and environmental regulation intensity on regional innovation performance has significant regional heterogeneity characteristics; it is mainly due to the regional differences of factor endowment, economic reform and economic development level in China’s provinces and regions.

Based on the empirical results and conclusions, this paper proposes three countermeasures and suggestions to improve the performance of regional innovation in China. Firstly, the central government should strengthen the institutional norms and incentive constraints on local government’s behavior choices. In the process of competition for FDI, local governments should fully consider the comparative advantages of regions and attach equal importance to FDI and local leading industries. Moreover, regional innovation should be improved by introducing appropriate technology and maintaining competitive advantage, but not at the expense of environment; in this way, the FDI flows will be consistent with regional factor endowment and sustainable economic and social development; furthermore, it can be helpful to maximize the technology spillover effect of FDI on regional innovation. When formulating environmental regulation policies, local governments must make proper use of environmental regulation tools such as pollutant discharge fees, user fees and emission trading fees; positive incentives must be provided for technological innovation of enterprises and improvement in their capabilities of pollution control and production efficiency.

Secondly, the transformation of local government competition should be guided from growth-oriented competition to green innovation-oriented competition; the local government’s competitive strategy of reducing the intensity of environmental regulation to compete for FDI should be gradually reversed. Furthermore, attention should be paid to promote the synergistic coupling between local government competition and environmental regulation intensity, and advocating “Green Innovation” [35]. The central government should introduce corresponding institutional arrangements, provide channels for promotion, clarify competition assessment indicators, and foster new competitive momentum of “green innovation” among local governments; at the same time, regional innovation performance in green development should also be improved. Local governments should actively implement the policies of the central government and focus on building regional innovation systems; they should also strengthen the support for regional innovation subjects by: improvement in the protection system of intellectual property rights and innovative achievements; creating new science and technology industrial parks; forming a regional innovation system of “government led, market participation and enterprise subject”.

Thirdly, considering the regional heterogeneity of local government competition and environmental regulation intensity, regional factor endowments and comparative advantages should be fully considered in the institutional arrangements to improve regional innovation performance. Thus, policy making goals should be achieved according to local conditions and by avoiding the “one size fits all” strategy of institutional arrangements. Since the eastern region of China has a high degree of marketization and advantages of economic development and factor endowment, the local governments in that region should focus on the international advanced technologies when competing for FDI; to establish linkage mechanisms in R&D cooperation, local governments should establish R&D centers through Chinese-foreign cooperation to encourage innovators. As far as environmental regulation is concerned, to achieve economic benefits, the government should focus on market-oriented environmental regulation tools and encourage enterprises to reduce pollutant emissions through technological innovation. In order to compete for FDI, local governments in the central and western regions should place emphasis on local innovation environment and carrying capacity. By improving the investment environment and policy support, they should introduce technology and management experience that has certain advantages over local markets but not great gaps. Efforts should be made to reduce duplication and import of low-level technology, and to eliminate vicious competition. As far as environmental regulation is concerned, it should be based on real development and environmental carrying capacity, and blind competition at the expense of the environment must be avoided. Last but not least, environmental regulation tools should be combined with government policies and market tools to strictly control the introduction of high-pollution and high-energy-consuming FDI.

## Figures and Tables

**Table 1:** Control variables selection and definition specification.

Variable Name	Definition Specification
Government intervention (gov)	Provincial fiscal expenditure/Provincial GDP
Development of Non-Agricultural Industries (fn)	Second and third industries as a share of GDP
Urbanization rate (urb)	The proportion of non-agricultural population in the total population, according to the permanent population
Financial development (fis)	Balance of loans of financial institutions at year-end/Provincial GDP
Human capital stock level (hum)	Average length of education for people over six years of age
Openness to the outside world (ope)	Total import and export trade of provinces/Provincial GDP

**Table 2:** Estimation results of the whole sample.

Variables	Model 1	Model 2	Model 3	Model 4
fdi	0.052 ***	−0.145 ***	0.133 ***	0.058 ***
	(7.48)	(−5.40)	(8.07)	(3.57)
er			0.452 ***	0.151 **
			(5.89)	(2.37)
fdi × er			−0.068 ***	−0.023 **
			(−5.41)	(−2.26)
gov				0.959 ***
				(8.05)
fn				−1.188 ***
				(−6.35)
urb				0.124
				(0.87)
fis				0.101 ***
				(3.47)
hum				−0.034 ***
				(−3.38)
ope				0.404 ***
				(12.35)
Constant term	0.317 ***	0.401 **	−0.222 **	1.119 ***
	(7.18)	(2.14)	(−2.18)	(6.26)
Regional effect	yes	yes	yes	yes
Time effect	yes	yes	yes	yes
Observation value	480	480	480	480

Notes: Numbers in parentheses are t-statistics for parameter estimation; ** for 5% level significant, *** for 1% level significant.

**Table 3:** Estimation of transformed explained variable.

Variables	Model 1	Model 2	Model 3	Model 4
fdi	0.816 ***	−0.145 ***	0.720 ***	0.340 ***
	(27.86)	(−5.40)	(11.31)	(4.74)
er			0.331	1.449 ***
			(0.98)	(4.92)
fdi × er			−0.077	−0.208 ***
			(−1.46)	(−4.89)
gov				0.384
				(0.65)
fn				−4.744 ***
				(−4.19)
urb				8.164 ***
				(13.73)
fis				0.454 ***
				(3.40)
hum				−0.069 *
				(−1.75)
ope				0.628 ***
				(6.61)
Constant term	−0.679 ***	0.401 **	−0.284	−2.398 ***
	(−3.68)	(2.14)	(−0.73)	(−2.71)
Regional effect	yes	yes	yes	yes
Time effect	yes	yes	yes	yes
Observation value	480	480	480	480

Notes: Numbers in parentheses are t-statistics for parameter estimation; * for 10% level significant, ** for 5% level significant, *** for 1% level significant.

**Table 4:** Estimation of transformed core explanatory variable.

Variables	Model 1	Model 2	Model 3	Model 4
fdi	1.182 ***	−1.562 ***	2.500 ***	1.363 ***
	(8.41)	(−3.64)	(9.26)	(3.68)
er			0.113 ***	0.053 ***
			(5.59)	(2.74)
fdi × er			−1.185 ***	−0.466 *
			(−5.26)	(−1.82)
gov				0.778 ***
				(5.44)
fn				−1.341 ***
				(−5.41)
urb				0.452 ***
				(3.13)
fis				0.177 ***
				(4.48)
hum				−0.049 ***
				(−3.45)
ope				0.247 ***
				(5.45)
Constant term	0.567 ***	1.393 ***	0.432 ***	1.485 ***
	(43.33)	(35.94)	(15.33)	(7.37)
Regional effect	yes	yes	yes	yes
Time effect	yes	yes	yes	yes
Observation value	480	480	480	480

Notes: Numbers in parentheses are t-statistics for parameter estimation; * for 10% level significant, ** for 5% level significant, *** for 1% level significant.

**Table 5:** Analysis of regional heterogeneity of sub samples.

Variables	Eastern Region		Central Region		Western Region	
	Model 3	Model 4	Model 3	Model 4	Model 3	Model 4
fdi	0.145 **	0.073 *	−0.116 ***	−0.140 ***	0.067 **	−0.011
	(2.54)	(1.67)	(−4.34)	(−3.02)	(2.05)	(−0.34)
er	1.916 ***	1.079 ***	−0.951 ***	−0.860 ***	0.411 ***	0.267 **
	(5.81)	(5.23)	(−5.28)	(−3.46)	(3.85)	(2.41)
fdi × er	−0.243 ***	−0.136 ***	0.150***	0.138 ***	−0.064 ***	−0.043 **
	(−5.48)	(−5.10)	(5.21)	(3.67)	(−3.07)	(−2.02)
gov		−0.431		0.499		0.761 ***
		(−1.51)		(0.91)		(3.47)
fn		−1.613 ***		−0.835*		2.165 ***
		(−5.52)		(−1.92)		(3.56)
urb		0.121		0.740 ***		0.526
		(0.41)		(3.06)		(1.40)
fis		0.190 ***		−0.126 *		−0.078
		(5.39)		(−1.79)		(−0.99)
hum		−0.006		−0.024		−0.089 ***
		(−0.95)		(−1.09)		(−3.17)
ope		0.208 ***		−0.569 *		0.655 **
		(5.81)		(−1.68)		(2.06)
Constant term	1.996 ***	2.540 ***	1.193 ***	2.006 ***	0.221	−0.869 *
	(4.69)	(7.41)	(7.25)	(6.76)	(1.37)	(−1.84)
Regional effect	yes	yes	yes	yes	yes	yes
Time effect	yes	yes	yes	yes	yes	yes
Observation value	176	176	128	128	176	176

Notes: Numbers in parentheses are t-statistics for parameter estimation; * for 10% level significant, ** for 5% level significant, *** for 1% level significant.
